# A support programme for caregivers of children with disabilities in Ghana: Understanding the impact on the wellbeing of caregivers

**DOI:** 10.1111/cch.12618

**Published:** 2018-09-27

**Authors:** Maria Zuurmond, Gifty Nyante, Marjolein Baltussen, Janet Seeley, Jedidia Abanga, Tom Shakespeare, Martine Collumbien, Sarah Bernays

**Affiliations:** ^1^ International Centre for Evidence in Disability London School of Hygiene and Tropical Medicine London UK; ^2^ University of Ghana Accra Ghana; ^3^ CBM East Africa Advisor Community Based Inclusive Development Kigali Rwanda; ^4^ London School of Hygiene and Tropical Medicine London UK; ^5^ PCG‐Health Coordination Office Accra Ghana; ^6^ Norwich Medical School University of East Anglia Norwich UK; ^7^ Faculty of Public Health and Policy London School of Hygiene & Tropical Medicine London UK; ^8^ Sydney School of Public Health, Sydney Medical School London School of Hygiene and Tropical Medicine London UK

**Keywords:** caregiver, cerebral palsy, child disability, evaluation, Ghana, wellbeing

## Abstract

**Background:**

Four fifths of the estimated 150 million children with disability in the world live in resource poor settings where the role of the family is crucial in ensuring that these children survive and thrive. Despite their critical role, evidence is lacking on how to provide optimal support to these families. This study explores the impact of a participatory training programme for caregivers delivered through a local support group, with a focus on understanding caregiver wellbeing.

**Methods:**

A qualitative longitudinal study was conducted to investigate the impact of a training programme, “getting to know cerebral palsy,” with caregivers on their wellbeing. Eighteen caregivers, from four districts, were interviewed up to three times over 14 months, to assess impact and the reasons for any changes.

**Results:**

Low levels of knowledge, high levels of stigma, physical and emotional exhaustion, and often difficult family relationships with social exclusion of the child and caregiver were common themes at the outset. Caregivers struggled to combine their caring and economic activities. This was exacerbated by the common absence of the father. Two months after completion of the training, their reported wellbeing had improved. The reasons for this were an improved understanding about their child's condition, positive attitudinal change towards their child, feelings of hope, and through the group support, a profound realisation that they are “not on their own.” While relationships within the family remained complex in many cases, the support group offered an important and alternative social support network.

**Conclusions:**

This study illustrates the many benefits of a relatively simple caregiver intervention, which has the potential to offer a mechanism to provide sustainable social support for caregivers and children with cerebral palsy. Any future programme needs to also address more structural issues, including stigma and discrimination, and strengthen approaches to family engagement.

**Key messages**

- Cerebral palsy is the most common cause of childhood physical impairment, and these children will often have complex care needs over their lifetime.
- A participatory training programme targeting caregivers in a support group setting can offer many important benefits, including improved understanding, confidence and self‐esteem, and a reduction in self‐blame resulting in improved care for the child.
- A support group appears to offer an important social safety net for caregivers often socially excluded in their own communities.
- There is an intersectionality of gendered caregiving, disability, and poverty, and a strengthened approach to more structural issues, including stigma and discrimination, and poverty reduction strategies is required.
- Families play an important role in caregiving, and we need to explore how we can better harness their capacity, including the engagement of fathers.

## 1 BACKGROUND

Globally, the most common cause of childhood physical impairment is cerebral palsy (Donald et al., 2014), estimated at 2 to 2.5/1,000 live births globally. Children with cerebral palsy may have complex care needs, due to sensory, intellectual and communication impairments, and possibly epilepsy, in addition to impaired motor function. And yet basic services available for them in low and middle income settings (LMICs) are frequently weak or absent (Donald et al., 2015). The new global child health agenda shifts the debate from survival to thriving, thus underlining the need to support both child and family (Colver, Fairhurst, & Pharoah, 2013; den Besten, Cornielje, Cornielje, & Botwey, 2016; Rosenbaum, 2011; Yousafzai, Lynch, & Gladstone, 2014).

Research in LMICs highlight that caregivers of children with disabilities frequently experience a range of difficulties: high levels of stress, anxiety, depression, and physical exhaustion, stigma, and discrimination often shaped by traditional beliefs and poverty (Bunning, Gona, Newton, & Hartley, 2017; Dambi, Jelsma, & Mlambo, 2015; Gona, Mung'ala‐Odera, Newton, & Hartley, 2011; Nakamanya, Siu, Lassman, Seeley, & Tann, 2014; Sandy, Kgole, & Mavundla, 2013; Tilahun et al., 2016). These can result in poorer caregiver quality of life, compared to caregivers of non‐disabled children (Dambi, Chivambo, Chiwaridzo, & Matare, 2015; Zuurmond, Mahmud, Polack, & Evans, 2015), as well having a negative impact on parenting (Barnett, Clements, Kaplan‐Estrin, & Fialka, 2003).

Despite high levels of need, there is a paucity of evidence on the efficacy of interventions for families of children with disability in LMICs (Donald et al., 2015; Donald, Samia, Kakooza‐Mwesige, & Bearden, 2014; Yousafzai et al., 2014). Parent focussed interventions have shown some positive benefits, some of which have been mediated through support groups, but these studies have largely been in high income settings (Masulani‐Mwale, Mathanga, Kauye, & Gladstone, 2018). Fewer studies in LMICs exist but have described some positive outcomes of training parent groups (Adams et al., 2011; McConachie et al., 2000; McConkey, Mariga, Braadland, & Mphole, 2000). With a view to strengthen future interventions, this study explores the impact of a community‐based parent support group training in Ghana, with a focus on understanding improvements in caregiver “quality of life” and “wellbeing.” These two terms are often used interchangeably (Camfield, Crivello, & Woodhead, 2009; Camfield & Skevington, 2008). Early theories on quality of life and wellbeing focussed on individual achievement of happiness and satisfaction (Andrews & Withey, 1976; Bowling, 1999, 2014) or on an individual's capability to pursue their goals (Deneulin & McGregor, 2010; Sen, 1993). The importance of social relationships and understanding of the collective character of human wellbeing came later (Deneulin & McGregor, 2010; Ruta, Camfield, & Donaldson, 2007), and our study draws upon this theoretical perspective.

For a holistic, relational, and contextual understanding of people's lives, we adopt a longitudinal qualitative design, drawing upon the socioecological model (Centers for Disease Control, 2015), to study the interplay of various factors at different levels of a social system (family, support group, and community) affecting changes in the caregiver.

## 2 METHODOLOGY

### 2.1 Intervention and theory of change

**Figure 1:**
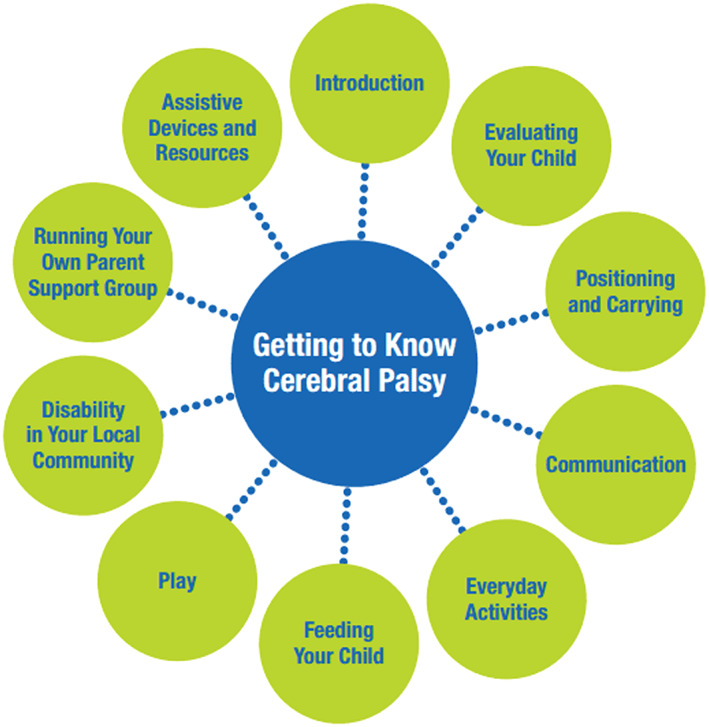
Outline of modules for parent support group training, available at http://disabilitycentre.lshtm.ac.uk/getting‐to‐know‐cerebral‐palsy/ [Colour figure can be viewed at wileyonlinelibrary.com]

Seventy‐five caregivers were invited to join a support group in one of the eight districts, with an average group size of 8–10 caregivers. Each group met monthly for an average of 4 hours, to cover the modules in “Getting to Know Cerebral Palsy” (LSHTM & Hambisela, 2013) as outlined in Figure 1.

The training was participatory and based on principles of adult learning theory (Knowles, 1984) to promote critical thinking, problem solving, and peer support. Core to the theory of change (ToC) was empowering the caregiver to improve care and support for their child, including an understanding of their rights, additionally, to share their learning with family members who in the West African setting have an important role in child‐rearing (Goody, 2005)

In total, 13 facilitators underwent a 1‐week master training and were paired to deliver the getting to know cerebral palsy training to each group. Each pair consisted of a physiotherapist/physiotherapy assistant and a primary health worker. Additionally, the facilitator/s made short monthly home visits to provide follow up on the training session and engage with other family members. Overall attendance at the programme was high with 92% of all families at endline having attended all of the training sessions.

### 2.2 Participant selection

Caregivers were identified through the community‐based screening programme for cerebral palsy, hospital records of children diagnosed with cerebral palsy in the last six months, and at one site additional screening was used to find sufficient local cases to run the group. Children had a confirmed diagnosis of cerebral palsy and were aged 18 months to 12 years, and caregivers had not attended any parent support group nor any regular physiotherapy with their child.

Four (out of eight) sites were then purposively selected for this qualitative study, ensuring a geographical spread, families of different socioeconomic status, and a mix of children according to gender, age (<6 and 6–12 years), and severity of cerebral palsy. Eleven families were selected in the first round, and following the death of three children, a further five families in the second round, and two more at endline. Interviews were conducted with 18 primary caregivers; seven were interviewed 3 times, five interviewed twice, and six interviewed once. Additionally, short opportunistic interviews were conducted with a secondary caregiver, at the time of the household interviews, in order to capture another perspective on caregiving within the family.

### 2.3 Research design

This study was part of a larger pre‐ and postintervention study. The qualitative longitudinal research was undertaken with between April 2015 and June 2016 (see Figure 1), in which we used repeat waves of data collection to facilitate an exploration of the household context and how lives are shaped by the economic, cultural, and social norms (Dornan & Woodhead, 2015) in order to have a richer understanding of how the training programme worked in practice. The research questions addressed in this paper are to explore (a) in what ways the intervention impacted upon the caregiver wellbeing and (b) the change process that caregivers engaged in, in order to inform future strengthening of the intervention.

### 2.4 Methods

**Figure 2:**
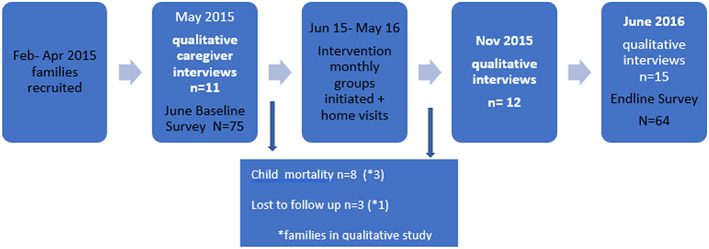
Flowchart showing research process [Colour figure can be viewed at wileyonlinelibrary.com]

Up to three in‐depth interviews were conducted with caregivers as outlined in Figure 2. The aim of having interviews midway was to capture the caregiver journey through the programme. All interviews followed a similar structure, exploring understanding of the condition, the health and wellbeing of the child and caregiver, and networks of support including access to services. The midline and endline interviews additionally focussed on exploring their experience of the parent group and home visits and how their experiences of the intervention may have changed over time.

Interviews were conducted by a lead interviewer (White‐British female MZ) and a local researcher (female Ghanaian GN), in four local languages with translation into English or into Twi. Interviews were conducted in the home setting; a “ladder of life” tool was used to explore caregivers' perceived quality of life, “0” being the first rung of the ladder and the worst possible life and “9” being the best top of the ladder and the best possible life (Crivello, Camfield, & Woodhead, 2009; den Besten et al., 2016). Photoelicitation, using photos taken of group activities, helped to reestablish rapport with the caregivers and stimulate more concrete discussions about the role of the parent support meetings and can be particularly useful in low‐literate groups (Banks, 1995; Morrow, 2001). In advance of follow‐up interviews, a short progress update was gathered about each child from the local facilitator, as well as group monitoring data, to tailor questions and probes to the family; for example, if the child was still malnourished, there was more probing about their experience with feeding and nutrition.

Ethics approval was obtained from the Noguchi Memorial Institute for Medical Research, University of Ghana and from the London School of Hygiene and Tropical Medicine. Informed written consent was obtained from all participating caregivers, with a signature or thumbprint. All children identified with malnutrition were referred for follow‐up, and the CBM child protection policy was adhered to.

### 2.5 Analysis

All interviews were translated into English and transcribed. An interpretive approach underpinned the data analysis (Creswell, 2013), with emphasis on the caregiver's perspective. Two key stages of the analysis were conducted: a thematic analysis across all the interviews at each of the three phases of interviewing, using a priori themes from the ToC, and then an iterative process of developing more themes and subthemes as interviews were compared. A second stage analysis of the same data was a biographical case study analysis of each caregiver, offering a more holistic overview of their journey over the 6‐ to 14‐month period, in line with guidance for longitudinal analysis (Creswell, 2013; J. Green & Thorogood, 2009). One researcher led the process (MZ), with ongoing discussion with the Ghana‐based researcher (GN). Data were managed using NVIVO 12 software.

## 3 FINDINGS

Key themes and subthemes were understanding of the condition with subthemes of diagnosis, traditional beliefs, acceptance, and hope; caring practices with confidence and behaviour change as subthemes; stigma with subthemes of exclusion/inclusion in different settings; mental health and peer support; and the cross‐cutting theme of poverty.

### 3.1 Our case study families

Fourteen caregivers were mothers, three grandmothers, and one a male cousin. Grandmothers were looking after the child because either the mother had died or had moved away. Most caregivers were traders or ran small businesses; the overall level of education was low, with only three caregivers having attended high school or tertiary education; and eight never having attended school. A socioeconomic index indicated that most families were economically very poor (See Table 1).

**Table 1:** Details of case study families

Caregiver relationship to child relation. M = mother, GM = GM	Caregiver occupation	Father living at home	Caregiver school level 0 = never attended, 1 = primary, 2 = junior high, 3 = senior high, 4 = tertiary	SES tertiles 1 = poorest
M	Trader	N	1	3
M	Trader	N—father moved out during the year, visits daily	3	3
GM	Manual work	N	0	2
M	Manual work	Dead	0	1
GM	Trader	N	1	2
M	Health worker	Y—father in prison at end line interview	4	2
M	Hairdresser	Y—works away from home regularly	2	1
M	Apprentice hairdresser	N—living separately in compound	0	2
M	Seamstress	N	0	2
M	Health worker	N—works away from home	4	3
GM	Trader (sells alcohol)	N	1	3
Cousin	Farmer	N	0	2
M	Trader	N	2	2
M	Farming	N	0	1
M	Farmer	N	0	1
M	Farmer	Y—polygamous, absent for last 7 months	0	1
M	Seamstress	Y—works away from home regularly	1	2
M	Apprentice seamstress	N	1	1

### 3.2 Understanding of the condition

At baseline, a major theme was very low levels of understanding the condition: Almost no child had been given a medical diagnosis, despite repeated hospital visits. Families had commonly spent considerable time and resources “seeking a cure” for their child:

I first took her to the hospital; the doctor said she was lazy because of her weight. We were then referred to the physiotherapy unit and we were given some days to attend. ……. I got sick of going to the hospital every day. (Code 3339 Grandmother, granddaughter of 2 years)

The absence of any medical diagnosis, combined with beliefs about disability being associated with witchcraft and family misfortune, meant that it was common to visit a range of traditional healers in seeking a cure, which included the use of various potent traditional medicines and sacrifices. Traditional beliefs were powerful and pervasive; a child with a disability was often not considered human. For example, in the south, the child could be described as “Asram”—a snake child or a river child. In the Frafra culture in the north, many children with a disability were described as “spirit children”: “Her body is dead. She is not a human being” (Code 3339).

The programme improved the understanding about their child's condition, including the “humanisation” of their child which came with this knowledge, and a new vocabulary to describe their child's condition which avoided a reliance on stigmatising terms:

Oh, it has helped us. Some felt the children are “water children” (nsuo ba) but we got to know that it is caused by a brain injury (adwen mu yare‐brain sickness) or when there is prolonged delivery (Code 1104 Mother with daughter of 18 months).

With improved understanding came a shift in attitudes which included greater acceptance of the child and hope. Overall, when explaining why their lives had improved, the caregivers commented on the positive impact of seeing changes in their child's development, even small changes, as it gave them hope. As well as hope felt from seeing their own child's development, there were “collective” benefits from seeing other children improve. Groups often needed time to become established, so these positive reflections emerged more towards the end of the year intervention.

Being in a group has helped me a lot because sometimes you see children with the same problem and it gives you hope … and I've see so many changes [in the group]. (Code 5560 to add Mother, son of 2 years).

### 3.3 Caring practice

Alongside the emotional and social strain of not having a medical diagnosis, the physicality of caregiving, associated with physical exhaustion was a recurrent theme at the baseline. Even when a caregiver lived within an extended family, they often received little support, so constant carrying and “backing” the child (carrying the child on their back) occurred, even when the mother was working. This became increasingly difficult for older and heavier children. Caregivers described frustration at not being able to put their child down as she would be crying. Some described how they were up in the night as their child did not sleep well. One mother wept as she explained the physical exhaustion of everyday caring for her 4‐year‐old son who had severe cerebral palsy: “My difficulty is my child he can't do anything for himself. I must do everything for him” (Code 55580).

By end line every caregiver reflected on both positive changes in their child and in their caregiving practice; changes in positioning of the child, in feeding practices, and understanding how to communicate with their child were popular changes described. In addition, several caregivers mentioned increased patience and less maltreatment of the child. This was noted as the most significant change observed by facilitators in their monthly monitoring of the groups.

When he used to eat, while crying, I used to beat him and when he was eating and turning here and there I didn't know how to take my time and feed him I just kept on feeding him. But when we went for the meeting they said whenever the child turns itself or is refusing to eat, we shouldn't do anything. (Code 3344 Mother, with 4‐year old son).

Increased confidence associated with improved understanding of how to care for their child, interlinked with less self‐blame for their child's disability, feeling valued in the group, and realising they had something to offer other mothers, appeared to link with improved self‐esteem.

### 3.4 Stigma and social inclusion/exclusion

At the baseline, interlinked with traditional beliefs was the stigma experienced by the child and caregiver in the home and in the community, including caregiver feelings of self‐stigma and shame. The effects were profound and were a major reason given for why fathers had left, family members were unwilling to care for the child, the child and caregiver faced exclusion in home and community life, and why the child may be neglected. All caregivers were initially tearful in describing their lives, exemplified by one mother, left by the husband, and who was told by a community member that it would be better if her daughter had died: “Anytime I remember I grow angry and even weep. My husband came to tell me that in his family, no one has ever given birth to a child like this, so what I've given him is a curse” (N02). Initially, some mothers were less open at talking about aspects of their experience of stigma and shame, while other family members, such as grandmothers, were more open.

Although family structures varied across sites, from extended families in one compound to smaller units in peri‐urban settings, caregiver isolation and social exclusion and an almost complete absence of a social support network were recurring themes. Fathers were almost completely absent from the household, whether because of separation or divorce, working away from home, and infrequent home visits (see Table 1). Asked whom she talked to when she had a problem, one mother replied:

I don't go anywhere I'm just in my room… I am not on good talking terms with my family members because of the problem [child's disability] and I am not close to the neighbours around, because of the child's condition too, so I do not draw near to them. (Code 5580 Mother, son of 4 years).

A widowed mother described how, despite being surrounded by her late husband's family, she had no one to leave the child with while she worked. Her daughter could “be lying down crying while people walking pass her … no one will touch her. They behave as if she is not human” (Code 3339).

By end line, the strongest recurrent theme was social inclusion from membership of the group “we have love for one another in the group,” and the value of “not the only one” and “not on their own,” which linked with reduced self‐blame for their child's disability. By the end of the year, the group was commonly described as “like a family.” The group offered an important support network that had been absent. The group offered each other support beyond the focus on their children.

The group was good because there was unity among us… Hum! I am alone sitting; I have no one to share my problem with… I know that I can share with them what my worry is, and there is understanding so they can support me. (case 3342 Mother with daughter of 12 years).

There was more limited progress in changes within the wider household dynamics. According to the ToC, the caregiver was expected to be the main agent of change within the family. There were examples of this, particularly within maternal families:

Before, I could not go to any place and leave the child behind. Anytime I went out, it was difficult for my mother to feed him. He used to vomit everything you feed him, but after the training, I also educated my mother and anywhere I go I come back to see him fed well. (9916 Single mum living with her own mother, son of 2 yrs).

However, it was still common for caregivers to describe limited sharing of knowledge and of the training resources with other family members, and for secondary caregivers to not be aware of the training content. Most notably, carers often did not share when living with the husband's family when family relationships related to the child remained difficult. For example, one mother with a 3‐year‐old son with severe cerebral palsy, after the intervention had stopped beating her son through frustration, his feeding and nutrition had improved and he no longer cried through the night. She also positioned him correctly rather than just laying him on the floor. She said that learning how to communicate with him “has touched my heart.” However, the family situation continued to deteriorate over the year: Her husband moved out while the mother‐in‐law looked for a second wife. His increasing lack of support meant that the mother struggled to pay for the child's health care, such as a hearing test. She did not feel able to share any learning within her husband's family.

The facilitator home visit was intended as one approach to engage with family members, and while all caregivers said they valued the fact that someone was prepared to visit them at their home, they commonly perceived it as “checking” that training was put into practice. The counselling element and engagement with other family members varied, but in general was limited, and monthly visits were sometimes prevented by a lack of transport and poor weather.

### 3.5 Caregiver mental health

While at baseline the strong theme was of poor caregiver mental health, there did seem to be some improvements by the end of the intervention. Improved knowledge, understanding they were not alone, with a reduction in self‐blame and feeling valued in the group, were factors which contributed to caregivers feeling emotionally better. Something as simple as a home visit could engender a sense of self‐worth for a caregiver:

[Before the training] No one was coming to us. It has always been the two of us here alone, my mum and I and we were always crying. Now, from the meetings, we have all learnt a lot and this happens no more. (Case:9916, Mother of son, 3 years old).

Other valued aspects of peer support from the group included peer learning, friendship, and a safe space to share experiences and offer psychosocial support, including encouragement.

Sharing of experiences is what I have enjoyed the most. Because when you have a problem you think your problem is greater than everyone else. We share ideas, sometimes there are things that they do with their children that we can do ourselves. (Code 5560).

The importance of the group also included the relationship with the facilitators, whose home visits were valued, and who commonly played a brokering role to help them access services, such as renewing their child's health card or navigating the disability benefits system, for example, by accompanying them to the relevant offices. In some cases, this appeared to generate some dependency on the facilitator.

### 3.6 Poverty

Poverty shaped the wellbeing of the majority of our caregivers and their ability to implement change from the training programme. The absence of fathers further contributed to a lack of resources, with no family accessing any social protection programmes at the outset of the training. Poor access to a livelihood was exacerbated if children were not in school, and/or the absence of family support, as described by one mother who carried her 5 year old daughter: “I put her on my back whilst I plant, when am tired I place her close to me so she can see me and not cry. What I could have done… When people need water for construction work, [I] carry her and get them the water.” (Code 3339).

“Because of him [her son] I can't even work, and his father has rejected us. My family members too are not taking care of me. And I have so many problems in the world and it is very hard and difficult.” (Code 5580).

By end line, as a result of improved understanding about their rights, caregivers had accessed or were in the process of accessing the national disability fund (District Assembly Common Fund). As their child improved, and with access to assistive devices, some time was freed up for work or household chores, such as a simple sitting chair that permitted a caregiver to leave a child comfortably while they worked at home, or they took with them to the marketplace. There was more confidence in being able to leave a child with a family member, but these gains were sometimes small, and the need for improved access to livelihoods remained a key caregiver request for improving the quality of their lives. However, poverty also impacted on the ability to implement some of the training, such as caregivers acquiring knowledge about nutritious food and how to feed their child. Some struggled to provide any food at all for the family, including the child with a disability.

Overall, the data showed that the training and support groups were valued, with a positive impact on caregiver wellbeing. At the same time, it illustrated how complex life was for caregivers, with reduced earnings due to caring responsibilities, community stigma reducing access to community support, and all coalescing and exacerbating each other.

## 4 DISCUSSION

In this paper, we explain why the overall caregiver quality of life improved as a result of engaging in the parent support training programme (Zuurmond et al., 2018). Changes were mainly at the level of the individual, most notably improvements in caregiver understanding about her child's condition, reduced feelings of shame and self‐stigma, and a shift in attitude towards their child, all of which was reflected in positive changes in care and support. The importance of building hope was an important component, promoting resilience in the face of long‐term stress, where resilience is defined as a the ability to harness resources to sustain wellbeing (Southwick, Bonanno, Masten, Panter‐Brick, & Yehuda, 2014). The process of parental adaptation and acceptance to having a child with a disability, important for parental wellbeing, has been documented in high income contexts (Barnett et al., 2003) and ties in with the apparent process of acceptance captured in this study, supported by meeting other caregivers.

We have also demonstrated the pervasive influence of societal stigma that shaped the outcomes in this Ghanaian context. The links between stigma and disability have been well evidenced (Van Brakel, 2006), and stigma has been has been shown to have complex and devastating effects on the health and welfare of individuals and families of children with disabilities, increasing stress and isolation (Bunning et al., 2017; S. E. Green, 2003; Nayar, Stangl, De Zalduondo, & Brady, 2014), and families of children with disabilities can play both a critical role in both perpetuating and reducing stigma (McConkey, Kahonde, & McKenzie, 2016). Stigma is a complex social construct (Goffman, 2009), and one useful type of categorisation of stigma is of “felt” or “self”‐stigma, which is about feelings of shame or fear of rejection, and enacted stigma describing overt rejection and discrimination (Gray, 2002). Our study suggests important changes in caregiver self‐stigma with positive changes in attitudes towards their child, which is notable given recent research in Ghana and Ethiopia with mothers of children with cerebral palsy and developmental disorders which documented high levels of caregiver self‐stigma and shame for their child's condition (Nyante, Carpenter, & Igo, 2017; Tilahun et al., 2016). Yet there was more limited change outside of the dyad, with persistent discrimination. This highlights the need for a strengthened approach to addressing stigma and discrimination in any future design, an improved understanding of the environment in which the children and their caregivers interact, and the role of structural issues (Kippax, Stephenson, Parker, & Aggleton, 2013). A recent study with children who were visually impaired made a similar argument for understanding the nested environment of the child and caregiver in order to strengthen programmes (Gladstone et al., 2017).

In terms of understanding pathways of change, this study illustrated that group membership appeared to offer a valuable social support network, in the face of caregiver social exclusion. The importance of such support networks for the care of children with disabilities is recognised (WHO 2011), yet few studies have sought to evaluate different types of support networks and how and why they work to improve caregiver and child outcomes, especially in LMICs. Self‐help groups for people with mental health conditions are increasingly used in low resource settings with evidence of their positive impact on both the person with the mental health condition and their caregiver (Cohen et al., 2012). Women's self‐help groups have resulted in improved maternal and child health outcomes because of the participatory approach to knowledge development, a more critical consciousness of the issues, community involvement, and acceptability of the approach (Morrison et al., 2010; Tripathy et al., 2010). Membership of a group can have positive and protective functions (Durkheim, 1952), and “collective agency” can support individual agency (Kippax et al., 2013). Establishing a local parent support group, in which the members have increased responsibility for sustaining the network, may possibly offer a more sustainable mechanism to address some of the ongoing support needs.

Finally, we illustrate the intersectionality of poverty, gendered caregiving and disability, and how this shapes the caregivers' wellbeing and may constrain their ability to implement changes within their family and household as a result of the training. This study contributes to the call for a more nuanced understanding of the relationship between poverty and disability (Groce, Kett, Lang, & Trani, 2011). In addition to the struggle to combine livelihoods and caregiving, our study illustrates how the impact of caring for a child with a disability has other significant consequences, such as the departure of a husband, or being socially excluded, which compounds poverty. These effects, though profound, are not easily captured within standardised socioeconomic measures.

These effects can coalesce to damage the social credibility of the caregivers that undermines their efficacy as agents of onward change within the community. So, this context arguably dilutes the time, capacity, and influence that these caregivers may have in persuading others to adjust their own attitudes and caregiving practices.

These issues point to a shortcoming in the intervention design, that of the focus was on the “empowered” caregiver/s as the main agent of change. In practice, this was problematic given the intersectionality of factors that affected their lives: poverty, stigma and discrimination, and gender, all of which serve to deplete many caregivers' resources, as well as challenging their credibility to influence. The overall low levels of caregiver education may be another factor. It points to any future intervention needing to explore additional ways to engage with family and community, with improved harnessing of external agents of change, community and faith‐based leaders, DPOs, and community health workers, such as the community‐based health planning and services in Ghana (Nyonator, Awoonor‐Williams, Phillips, Jones, & Miller, 2005). It may also be why external facilitators need to play an important brokering role at the start of such a programme, with a shift to greater parental leadership and building collective action, ensuring that facilitators have the skills in participatory approaches to facilitate this.

There are several limitations to this study. There was no follow‐up after the intervention had finished, which would have added considerable value in understanding how sustainable these effects were. Collective action by the groups, including public engagement activities, occurred after the data collection phase, and it would have been of value to capture and understand that contribution, in any future study. The intervention is highly dependent on the quality of the facilitators and their ability to establish rapport with the caregivers. Future evaluations of similar interventions would benefit from including more observations of the group activities and home visit, including focusing on the relationships between facilitators and caregivers. Two thirds of our sample were 4 years old or below, and future research should explore the views of older children on the quality of their lives as a result of engagement in the programme.

## 5 CONCLUSION

This study highlights several important benefits from a relatively simple intervention where caregivers join a local support group and engage with a participatory training programme, including positive changes in caregiver practices and peer‐based social support. Our study has shown that this model has considerable potential and could feasibly be implemented with other families affected by other disabilities. However, we propose ways that the model could be further strengthened. The use of caregivers as facilitators or co‐facilitators in the training may go some way to strengthening their role as agents of change. Such an approach is currently underway in Uganda for children with neurodevelopmental conditions (Martin et al., 2017) and in Brazil with children with Zika (Kuper, Smythe, & Duttine, 2018). The intervention is currently narrowly focussed on the relationship between the dyad of caregiver and child. To pursue a more transformative agenda, then the model needs to more effectively engage those around the caregiving dyad, including the broader family, husbands' relatives, and the community. This is vital to address stigma, discrimination, and ameliorate the effect of disability on household poverty. An extended intervention could also be adapted for the wider community: local personnel and leaders, such as teachers, primary health‐care workers, and faith leaders, recognising the need to reduce the dependency on the caregiver as the agent of change and underlining of the importance of applying a gendered lens so there is not an additional burden to already overstretched female caregivers.

## CONFLICT OF INTERESTS

The authors declare no conflict of interest.
